# Roles of the Skp2/p27 axis in the progression of chronic nephropathy

**DOI:** 10.1007/s00018-012-1232-x

**Published:** 2012-12-20

**Authors:** Sayuri Suzuki, Naro Ohashi, Masatoshi Kitagawa

**Affiliations:** 1 Department of Molecular Biology, Hamamatsu University School of Medicine, 1-20-1 Handayama, Hamamatsu, 431-3192 Japan; 2 Internal Medicine 1, Hamamatsu University School of Medicine, Hamamatsu, Japan

**Keywords:** Ubiquitin-proteasome, Ubiquitin ligase, Chronic nephropathy, p27, Skp2

## Abstract

S-phase kinase-associated protein 2 (Skp2) is an F-box protein component of the Skp/Cullin/F-box-type E3 ubiquitin ligase that targets several cell cycle regulatory proteins for degradation through the ubiquitin-dependent pathway. Skp2-mediated degradation of p27, a cyclin-dependent kinase inhibitor, is involved in cell cycle regulation. Tubular epithelial cell proliferation is a characteristic feature of renal damage that is apparent in the early stages of nephropathy. The p27 level is associated with the progression of renal injury, and increased Skp2 expression in progressive nephropathy is implicated in decreases of p27 expression. In Skp2^−/−^ mice, renal damage caused by unilateral ureteral obstruction (UUO) was ameliorated by p27 accumulation, mainly in tubular epithelial cells. However, the amelioration of UUO-induced renal injury in Skp2^−/−^ mice was prevented by p27 deficiency in Skp2^−/−^/p27^−/−^ mice. These results suggest that the Skp2-mediated reduction in p27 is a pathogenic activity that occurs during the progression of nephropathy. Here, we discuss the roles of the Skp2/p27 axis and/or related signaling pathways/components in the progression of chronic nephropathy.

## Introduction

Cell proliferation is a fundamental biological mechanism that involves transit through the cell cycle. It is regulated by a network of proteins including cyclins, cyclin-dependent kinases (CDKs) [1], and CDK inhibitors (CKIs) [2]. The CKI p27^*Kip1*^ (p27) is a negative regulator that halts progression from the G1 phase to the S phase in the cell cycle. p27 is abundantly expressed in most normal quiescent cells, whereas its level declines when cells are stimulated to proliferate in response to mitotic stimuli, allowing progression to the S phase [3, 4]. The ubiquitin-proteasome pathway for protein degradation plays an important role in regulating the abundance of cell cycle regulatory proteins [5, 6]. Protein degradation via the ubiquitin-proteasome pathway is rapid and substrate-specific, which is consistent with its role in controlling fluctuations in the intracellular concentrations of cyclins and CKIs. S-phase kinase-associated protein 2 (Skp2) is an F-box protein component of the Skp/Cullin/F-box (SCF)-type E3 ubiquitin ligase that plays important roles in regulating the progression to the S phase. p27 is phosphorylated at threonine residue 187 (Thr187) by CDK2/cyclin E. The SCF/Skp2 complex interacts with phosphorylated p27 to promote p27 degradation through the ubiquitin-proteasome pathway [7, 8]. The cdc kinase subunit 1 (Cks1) is an essential cofactor for SCF/Skp2 ubiquitin ligase to ubiquitylate p27. Cks1 recognizes and binds to Thr187-phosphorylated p27 and induces rigid binding between Skp2 and p27 [9, 10]. p27 is stabilized in Skp2-deficient mice [11]. Therefore, proteasomal ubiquitin-dependent degradation of p27 is specifically controlled by the SCF/Skp2/Cks1 complex.

In the kidney, cell proliferation is thought to represent a central response to renal injury culminating in end-stage renal disease caused by the progression of tubulointerstitial fibrosis [12]. Disruption of the balance between cell proliferation and apoptosis leads to unchecked apoptosis of damaged tubular epithelial cells resulting in progressive tubular cell loss, renal tubular atrophy, and advanced interstitial fibrosis [13].

## Unilateral ureteral obstruction (UUO) and anti-thymocyte serum (ATS) are models of chronic nephropathy

**Fig. 1:**
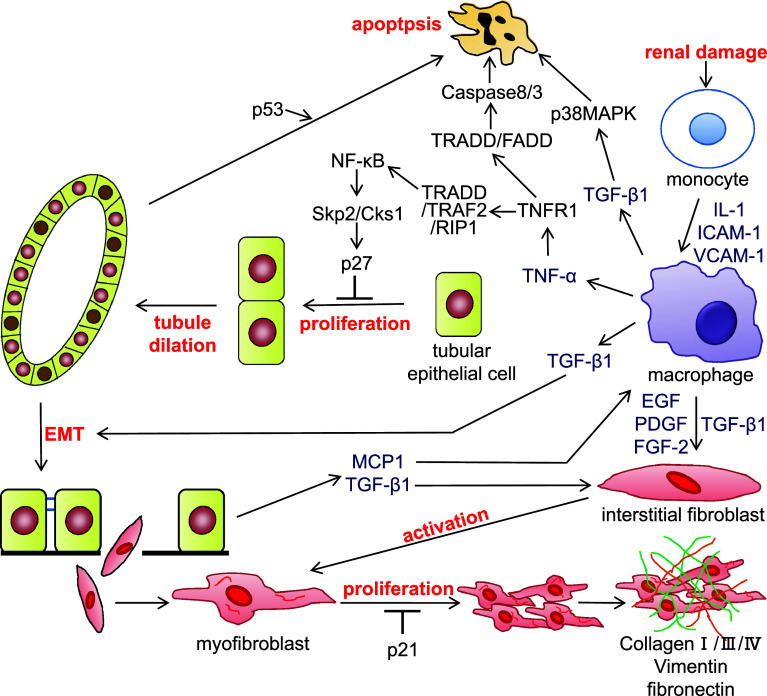
The signal transduction pathways involved in the progression of chronic nephropathy. Following renal damage, infiltrated macrophages in the tubulointerstitium release cytokines such as tumor necrosis factor-α (TNF-α) and transforming growth factor-β1 (TGF-β1). TNF-α binds to TNF receptor 1 (TNFR1) and forms a complex with TNFR-associated death domain (TRADD), TNF associated factor 2 (TRAF2), and receptor interaction protein 1 (RIP1). This complex activates nuclear factor (NF)-κB, which induces Skp2 and Cks1. Upregulation of Skp2/Cks1 promotes p27 degradation in tubular epithelial cells, allowing proliferation of tubular epithelial cells and tubule dilation following the increase of tubular epithelial cell number. The tubular epithelial cells undergo epithelial-mesenchymal transition (EMT) by stimulation of TGF-β1, and the resulting fibroblasts migrate to the tubulointerstitium. Cytokines including TGF-β1 activate fibroblasts; activated myofibroblasts produce extracellular matrix components, such as collagen, vimentin, and fibronectin. Meanwhile, TNF-α and TGF-β1 induce tubular epithelial cell apoptosis. *IL-1* interleukin-1, *ICAM-1* intercellular adhesion molecule-1, *VCAM-1* vascular cell adhesion molecule-1, *EGF* epidermal growth factor, *PDGF* platelet-derived growth factor, *FGF-2* fibroblast growth factor-2, *FADD* Fas-associated death domain protein, *MAPK* mitogen-activated protein kinase

UUO is a widely used model of kidney disease associated with progressive tubulointerstitial damage. This method has been used to identify many of the cellular and molecular events that occur during the progression of renal fibrosis, including events associated with cell proliferation and apoptosis [14–16]. UUO kidneys show elevated expression levels of monocyte chemoattractant protein-1 (MCP-1), vascular cell adhesion molecule-1 (VCAM-1), and intercellular adhesion molecule-1 (ICAM-1), which promote monocyte infiltration and kidney inflammation [17, 18]. It is generally believed that renal tubule dilation occurs as a result of increased hydrostatic pressure following obstruction. However, it was also reported that decreases in renal blood flow and the glomerular filtration rate both promote macrophage invasion into the renal interstitium. The infiltrated macrophages release various cytokines, including TNF-α [19]. The cytokine signals and hydrostatic pressure may act collaboratively to stimulate epithelial cell proliferation, which results in an increased number of tubular epithelial cells. We previously reported that tubule dilation is correlated with the increase in number of epithelial cells and enhanced tubular epithelial cell proliferation in the obstructed kidney [20]. Taken together, these results suggest that hydrostatic pressure and tubular epithelial cell proliferation are involved in tubule dilation. Renal tubular epithelial cell proliferation increases significantly and renal tubules start to dilate at 3 days after UUO [20, 21]. The extent of tubule dilation is related to the progressive increase in tubular epithelial cell number caused by proliferation. This process ultimately results in the fracture of the tubular basement membrane of the dilated renal tubules. In damaged kidneys, tubular epithelial cells trans-differentiate into mesenchymal cells that express α-smooth muscle actin (α-SMA) in response to kidney inflammation. These cells enter the tubular interstitium through the broken tubular basement membrane [22, 23]. The trans-differentiated tubular epithelial cells further differentiate into myofibroblasts (i.e., fibroblasts expressed α-SMA) in the interstitium. Concurrently, macrophages in the renal interstitium release several cytokines, including epidermal growth factor (EGF), platelet-derived growth factor (PDGF), and fibroblast growth factor-2 (FGF-2), which activate fibroblasts. The interstitial myofibroblasts undergo hyperproliferation because of their high cell responsiveness, resulting in irreversible progression of renal interstitial fibrosis (Fig. 1). There are many reports of establishing UUO in knockout mice and the roles of many cell cycle-related molecules in renal damage have been investigated in UUO kidneys [24].

Another experimental model of chronic progressive glomerulonephritis can be induced in rats by repeated injections of ATS. In this model, irreversible glomerulosclerosis and tubulointerstitial fibrosis are induced after the second ATS injection and are associated with a gradual decline of renal function [25–27]. Alternatively, chronic renal failure can also be studied in the 5/6 nephrectomy model [28, 29] and in diabetic nephropathy [30, 31].

## Signal transduction pathways involved in renal damage

### Transforming growth factor-β1 (TGF-β1)/Smad pathway

TGF-β1 is a multifunctional signaling protein that regulates cell cycle, apoptosis, differentiation, and extracellular matrix accumulation [32]. TGF-β1 also has a significant role in the progression of renal fibrosis in clinical and experimental kidney diseases [25, 33]. Following the onset of nephropathy, TGF-β1 is released from macrophages in the damaged renal interstitium and influences the tubular epithelial cells. The damaged tubular epithelial cells also release TGF-β1, which exacerbates renal damage. TGF-β1 was also reported to stimulate the epithelial-mesenchymal transition (EMT) [34–36]. Finally, tubular epithelial cells that acquire a fibroblastic phenotype via EMT migrate into the interstitium, probably through the ruptured tubular basement membrane. TGF-β1 also promotes the differentiation of interstitial fibroblasts to myofibroblasts and their production of extracellular matrix [37, 38]. The accumulation of extracellular matrix in the tubulointerstitium and in the glomerulus is also stimulated by TGF-β1. Conversely, TGF-β1 promotes apoptosis of tubular epithelial cells via a p38 mitogen-activated protein kinase-dependent mechanism [39, 40]. Overall, upregulation of TGF-β1 contributes to EMT during renal fibrosis and apoptosis, and it induces the progression of nephropathy.

In terms of the TGF-β1 signaling pathway, Smad proteins play important roles as signal transducers downstream of TGF-β1 receptors [41, 42]. TGF-β1 binds to the TGF-β type II receptor, which recruits and phosphorylates the TGF-β type I receptor, ALK5. In turn, ALK5 phosphorylates Smad2 and Smad3, which then bind to Smad4 [43, 44]. The resulting complexes can then enter the nucleus [45–47]. Another Smad, Smad7, has an inhibitory role in the TGF-β1 signaling pathway [48]. It was also reported that chronic progressive renal injury can be suppressed by inhibiting the TGF-β/Smad axis using an anti-TGF-β antibody [27]. Although TGF-β1 signaling is also mediated by ALK1, another TGF-β type I receptor that phosphorylates Smad1/5 [49], little is known about the roles of the ALK1/Smad1/5 pathway in renal injury. It was also suggested that TGF-β promotes translocation of Skp2 into the nucleus, where it is degraded by the anaphase-promoting complex/cyclosome (APC/C)-Cdh1 E3 ligase. In addition, TGF-β decreases Cks1 mRNA expression, which allows p27 to accumulate following G1 arrest [50–52]. Taken together, these findings indicate that TGF-β is an important upstream signal that regulates the Skp2/p27 axis.

### Tumor necrosis factor-α (TNF-α)/nuclear factor (NF)-κB pathway

TNF-α is a multifunctional cytokine that induces a wide range of cellular responses, including proliferation, differentiation, and activation of apoptosis [53]. TNF-α is produced by activated macrophages, and it stimulates the proliferation and apoptosis of renal tubular epithelial cells and interstitial cells in renal injury [54–56]. TNF-α binds to two different TNF receptors (TNFR), type 1 and type 2 receptors [57, 58]. On binding of TNF-α to TNFR1, TNFR1 recruits TNFR-associated death domain (TRADD) as an adaptor protein thorough death domain within 2 min. In turn, TRADD serves as an assembly platform protein to arborize TNFR1 signaling between apoptosis and anti-apoptosis/proliferation. TRADD recruits Fas-associated death domain protein (FADD) to its death domain and activates the Caspase-8/-3 cascade to induce apoptosis [59, 60]. TRADD also recruits TNF-associated factor 2 (TRAF2) and receptor interaction protein (RIP), leading to the activation of NF-κB, which has anti-apoptotic effects [57, 61]. It has been reported that the TNFR1/TRADD/TRAF2/RIP complex is produced more quickly than the TRADD/FADD complex because of the antagonistic effects of the TNFR1/TRADD/FADD on apoptosis signaling pathways.

TNF-α can also bind to TNFR2, which recruits TRAF2 and activated NF-κB [62]. However, binding of TNF-α to TNFR2 promotes TRAF2 degradation through the ubiquitin-dependent proteasome pathway, resulting in the suppression of NF-κB activation by inhibition of TRADD/TRAF2/RIP complex formation. In addition, TNF-α decreases TRADD protein levels by enhancing its ubiquitin-dependent degradation in obstructive renal damage [20]. In the kidneys, it was reported that renal damage caused by cisplatin was less severe in TNFR2-deficient mice than in TNFR1-deficient mice [57]. However, renal damage in UUO mice was less severe in TNFR1-deficient mice than in TNFR2-deficient mice [63]. It was also reported that a reduction of TRADD inhibits TNFR1 signaling and that TNFR1-mediated TNF-α signaling may transfer to TNFR2 signaling in UUO mice [21]. Another report revealed that the two TNFRs may act collaboratively to regulate signal transduction [64, 65]. However, it has been unclear how TNFR2 regulates the TNF-α signaling pathway until now.

The transcription factor NF-κB, a downstream factor of TNF-α, is activated in renal damage and controls the activation of many genes related to inflammation [66, 67]. NF-κB is an inductive homo- or heterodimeric transcription factor composed of the Rel family members of DNA-binding proteins, including p50/p105 (NF-κB1), p52/p100 (NF-κB2), RelA (p65), RelB, and c-Rel [68]. Activated NF-κB behaves as an important regulator of inflammation and immune responses by mediating the expression of pro-inflammatory genes, including cytokines, chemokines, growth factors, and adhesion molecules, which are implicated in the progression of renal inflammatory disease [69, 70]. The downstream targets of NF-κB are also important regulators of cell proliferation. For example, the IκB-inducing kinase (IKK)-regulated signaling pathway accelerates cell proliferation. Furthermore, IKK-α, an essential component of the NF-κB pathway, affects many physiologic activities in both healthy and disease states [71], including mammary epithelial cell proliferation [72]. In renal injury, NF-κB stimulates tubular epithelial cells and fibroblasts, and induces their proliferation and differentiation, which ultimately promote the progression of renal fibrosis [73]. It was reported that the NF-κB pathway regulates Skp2 expression [74, 75]. As described below, we have suggested that TNF-α stimulates Skp2 and Cks1 mRNA expression via the NF-κB pathway in chronic nephropathy [76]. Therefore, TNF-α is likely to participate in Skp2/Cks1-dependent degradation of p27 as a precipitating factor of chronic nephropathy.

## Role of the Skp2/p27 axis in the progression of renal damage

### Skp2

**Fig. 2:**
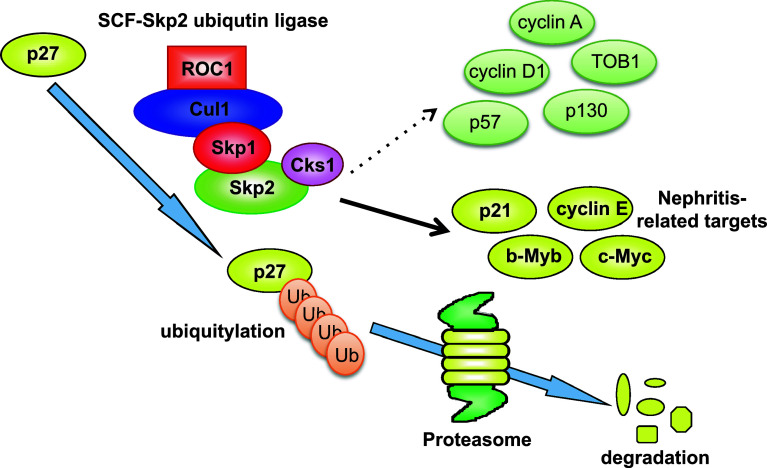
The mechanism of p27 degradation by Skp2 E3 ubiquitin ligase. SCF (Skp1/Cul1/Skp2 as F-box) ubiquitin ligase induces p27 degradation by a proteasome-dependent pathway. Cks1 is an essential cofactor for p27 degradation by SCF/Skp2 that induces rigid binding between Skp2 and p27. Conversely, Skp2 has multiple targets and may also regulate p21, cyclin E, b-Myb, and c-Myc protein levels in unilateral ureteral obstruction (UUO). However, Skp2 did not affect the regulation of cyclin A, cyclin D1, TOB1, p57, or p130 in UUO kidneys

The SCF/Skp2 ubiquitin ligase complex targets several important regulator proteins that control the cell cycle, including p27, p21, p57, cyclin E, cyclin A, and cyclin D1 [77], by promoting their degradation via the ubiquitin proteasome-dependent pathway. In this way, Skp2 ubiquitin ligase promotes cell cycle progression to the S-phase by stimulating the degradation of negative cell cycle regulators, such as the CKI p27 [7, 8, 78] (Fig. 2). Moreover, it has been reported that Kip1 ubiqutination-promoting complex (KPC) [79] and Pirh2 [80] act as E3 ligases for p27, whereas it has not been clarified whether p27 is accumulated in their knockout mice. In human cancers, it was demonstrated that Skp2 overexpression stimulates the degradation of p27, indicating that Skp2 overexpression facilitates accelerated tumor growth and malignant potential [77]. However, the proteins that are targeted by Skp2 for degradation in specific biological processes or diseases have not been fully characterized.

**Fig. 3:**
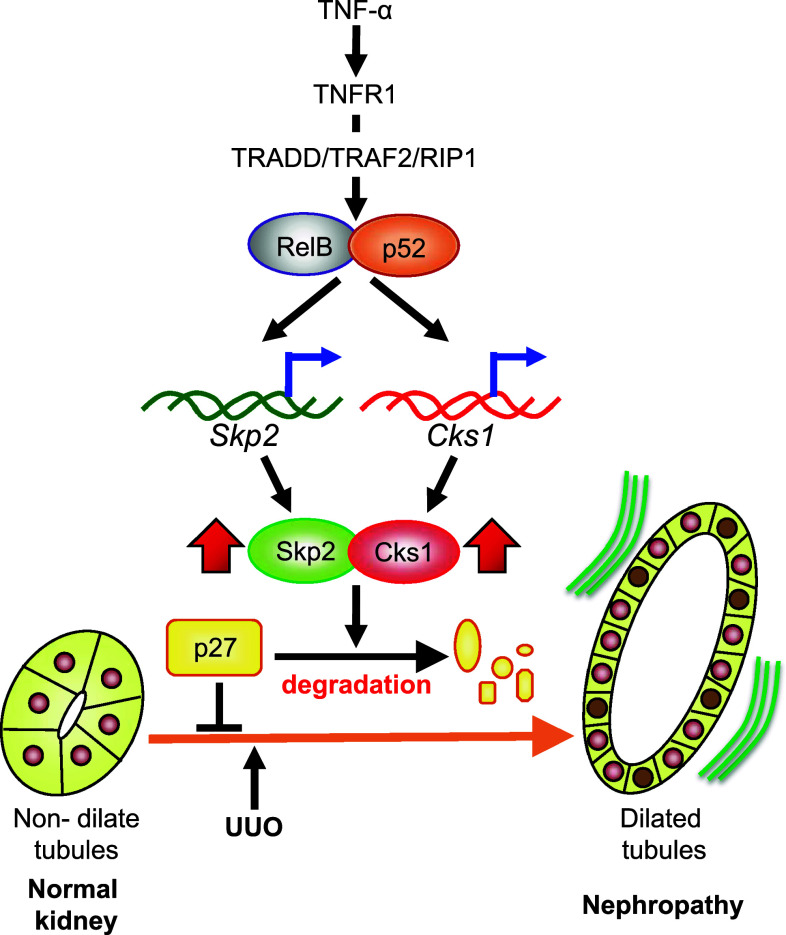
Skp2/Cks1 is induced by the TNF-α/NF-κB signaling pathway in nephropathy. In normal kidneys, tubular epithelial cells highly express p27 in the quiescent phase of the cell cycle. Following renal damage, TNF-α activates RelB/p52, known as NF-κB, via TNFR1. The activated RelB/p52 complex induces the expression of both Skp2 and Cks1 in the nucleus. The induced Skp2/Cks1 degrades p27 in tubular epithelial cells in UUO kidneys, allowing tubular epithelial cell proliferation to increase [76]. Tubular dilation occurs as a result of the increase in the tubular epithelial cell number and ultimately leads to progressive nephropathy [20]

We previously reported that Skp2 mRNA expression was increased in UUO kidneys in the early stages of renal damage and that the progression of tubulointerstitial fibrotic damage in UUO kidneys is attenuated in Skp2-deficient mice [20]. Furthermore, as described above, the mRNA and protein levels of Skp2 were increased in the ATS model of chronic nephropathy in rats [76]. It was reported that the NF-ĸB signaling pathway regulates the Skp2 promoter in cultured cells [74, 75]. TNF-α was reported to enhance mRNA expression of Skp2 in a normal rat epithelial kidney cell line (NRK) but not in control cells, which suggests that TNF-α facilitates the induction of Skp2 in nephropathy. In damaged kidneys, exposure to TNF-α significantly increased in cytoplasm of tubular epithelial cells. RelB and p52 proteins are known as NF-κB, and they are mainly seen in the nuclei of tubular epithelial cells. Skp2 is also expressed in the nuclei of tubular epithelial cells, similar to RelB and p52. Skp2 and RelB are colocalized in renal damage [76]. These data suggest that Skp2 is induced by the TNF-α/RelB/p52 signaling pathway in the early stages of renal injury and facilitates ubiquitin-dependent degradation of p27 in tubular epithelial cell proliferation and in the progression of chronic nephropathy (Fig. 3).

### Cks1

Cks1 is an essential cofactor for ligation of ubiquitin to p27. It recognizes Thr187-phosphorylated p27 and is essential for the rigid binding between p27 and Skp2 that results in Skp2-mediated degradation of p27 [9, 10]. We previously reported that the mRNA and protein levels of Cks1 are increased in the early stages of renal damage [76]. Cks1 protein is mainly localized in the nuclei and to a lesser extent in the cytoplasm of tubular epithelial cells. Similar to Skp2, Cks1 colocalizes with RelB in the nuclei of tubular epithelial cells. These results suggest that Skp2 and Cks1 collaboratively promote p27 degradation via the ubiquitin proteasome pathway and induce tubular epithelial cell proliferation in the early stages of renal damage, resulting in tubular dilation in chronic nephropathy. The mRNA level of Cks1 is also significantly upregulated in TNF-α-stimulated NRK cells. We also reported that a sequence (GGGACTTCC) in the rodent Cks1 promoter is similar to the putative NF-κB element (GGGACTTTCC) at nine of the ten nucleotides. Therefore, it is seems likely that the TNF-α/NF-κB signaling pathway promotes the transcription of both Skp2 and Cks1 in renal injury [76].

### p27

The CKI p27 is an important regulator of cell proliferation that negatively regulates the behavior of CDKs in the cell cycle [81, 82]. p27 is abundantly expressed in most normal quiescent cells, but its level decreases during progression to the S phase in response to a proliferative/mitotic stimulus [3, 4]. In vitro studies have shown that an experimental decrease of p27 protein enhances the proliferative response to mitogens [83, 84], while forced overexpression of p27 protein inhibits cell proliferation [4]. Additionally, p27 is destabilized in many types of human cancer, which is implicated in the aggressiveness and poor prognosis of tumors [77, 85–87]. The protein level of p27 is controlled transcriptionally and by proteolytic degradation of p27 protein via the ubiquitin-proteasome pathway. p27 is phosphorylated on Thr187 by CDK [9, 10], and Thr187-phosphorylated p27 is a specific target for the SCF/Skp2/Cks1 complex to induce its ubiquitin-dependent degradation [7, 11]. This is consistent with observations that Skp2-deficient mice and/or Cks1-deficient mice exhibit cellular accumulation of p27 and a small body size compared with wild-type mice [9, 10].

In normal kidneys, p27 is expressed in most tubular epithelial cells to maintain their quiescent status. The level of p27 protein decreases rapidly in UUO kidneys, allowing proliferation of tubular epithelial cells and tubule dilation in the early stages of nephropathy. The mRNA and protein levels of p27 are subsequently upregulated in UUO mice [88, 89]. It was reported that renal tubular epithelial cell proliferation and apoptosis are markedly increased in the obstructed kidney of p27^−/−^ mice [90]. Additionally, the magnitude of p27 protein upregulation in obstructed kidneys is greater in Skp2^−/−^ mice than in Skp2^+/+^ mice. In the UUO kidneys of Skp2^−/−^ mice, tubular epithelial cell proliferation is inhibited by the accumulation of p27, preventing an increase in tubular epithelial cell number. Furthermore, apoptosis and tubulointerstitial fibrosis are markedly attenuated in the obstructed kidneys of Skp2^−/−^ mice [20]. It is well known that renal fibroblast activation and proliferation are involved in the progression of chronic kidney disease [19]. We also reported that UUO stimulates renal interstitial cell proliferation and significantly increased the number of interstitial cells in the UUO kidney [20]. The enhanced interstitial cell proliferation and the increase in number of α-SMA-positive myofibroblasts were partially inhibited by Skp2-deficiency. p21 is the critical negative regulator of interstitial fibroblast proliferation [91]. We have shown that p21 accumulation in UUO kidneys is moderately enhanced by Skp2 deficiency [20]. In addition, the accumulation of p21 and p27 as a result of proteasome inhibition is associated with inhibition of interstitial fibroblast proliferation [92]. Therefore, p21 and p27 are negative regulators of interstitial cell proliferation while upregulated Skp2 in the UUO kidney enhances their degradation to promote interstitial fibroblast proliferation and myofibroblast formation as critical stages in the EMT. Taken together, these results suggest that Skp2 has important roles in the control of p27 and p21 in the kidney. In addition, Skp2, as induced by renal damage, promotes the proliferation of tubular epithelial cells and interstitial fibroblasts by enhancing the degradation of p27 and p21. Although further investigation is required to determine whether renal function was recovered by Skp2 deficiency, the histopathological features of Skp2^−/−^ UUO kidney were apparently improved compared with the WT UUO kidney. Many other studies have demonstrated increased p27 expression in other models of renal disease, including diabetic nephropathy [30, 31] and cisplatin-induced acute renal failure [93]. In kidney cells, mesangial cells (MC) play a key role in glomerular hypertrophy in early diabetic nephropathy [94] by secreting extracellular matrix proteins that contribute to the development of glomerulosclerosis. Increased p27 expression in the glomerulus causes proliferation arrest and hypertrophy of MCs during early diabetic nephropathy. p27 is also highly expressed in the normal quiescent rat glomeruli, but its expression decreases in proliferating MCs in the ATS model of nephropathy [95]. The p27 expression level returns to the basal level after the resolution of MC proliferation [96]. Podocyte proliferation is also markedly increased in association with glomerulonephritis in p27^−/−^ mice [90]. These data indicate that p27 regulates the proliferation of various types of renal cells, and its upregulation stops excessive renal cell proliferation to protect cells and tissues from inflammatory injury.

### Renal damages in Skp2^−/−^p27^−/−^ mice

Unlike the marked amelioration of renal injury associates with renal accumulation of p27 in tubular epithelial cells in Skp2^−/−^ mice, Skp2^−/−^/p27^−/−^ double knockout mice show marked progression of tubular dilatation as a result of the enhanced tubular epithelial cell proliferation that occurs through the loss of p27 [97]. Notably, the tubular epithelial cell number in UUO kidneys is much greater in Skp2^−/−^p27^−/−^ mice than in wild-type mice. Furthermore, interstitial cell proliferation in UUO kidneys is also greater in Skp2^−/−^p27^−/−^ mice than in Skp2^−/−^ mice. The expression levels of vimentin, α-SMA, type I collagen, and fibronectin, components of the extracellular matrix, are significantly decreased in the UUO kidneys of Skp2^−/−^ mice. While extracellular matrix production and macrophage infiltration are more pronounced in these mice, tubulointerstitial fibrosis progresses more in Skp2^−/−^p27^−/−^ mice compared with Skp2^−/−^ mice [20, 91]. These results suggest that Skp2 may regulate extracellular matrix synthesis by modulating p27 expression/activity in renal diseases. Taken together, these results indicate that the ameliorative effects of Skp2 deficiency following UUO are canceled by p27 deficiency in Skp2^−/−^p27^−/−^ mice. As described above, it has been reported that proliferation is inhibited, and that the expression of p21 and p27 is increased by proteasome inhibitors in two nasal fibroblast cell lines. In these cell lines, treatment with a proteasome inhibitor suppressed fibrosis together with reduced MCP-1 production and TGF-β- and TNF-α-induced collagen mRNA expression. Moreover, the inflammatory response in fibroblasts is inhibited by suppression of IL-1β-/TNF-α-induced NF-κB activation and IL-1β-induced IL-6/8 production [92]. These results suggest that the accumulated p21 and p27 in fibroblasts can inhibit tissue inflammation and progressive fibrosis. In the UUO kidneys of Skp2^−/−^ mice, extracellular matrix production, inflammation, and renal fibrosis may be ameliorated by p27 accumulation.

In addition to p27, Skp2 targets several other proteins that control the cell cycle, including p21, p57, cyclin E, cyclin A, and cyclin D1, for degradation via the ubiquitin-dependent proteasome pathway. Interestingly, the protein levels of other Skp2 targets, including p57, p130, TOB1, cyclin A, and cyclin D1, in UUO kidneys were not significantly increased in Skp2^−/−^ mice compared with wild-type mice. Although the levels of p21, c-Myc, b-Myb, and cyclin E, in the UUO kidneys were slightly increased in Skp2^−/−^ mice, the magnitudes of the increments did not reflect the accumulation of p27 [97]. These findings suggest that p27 is the main target of Skp2 and that the reduction in p27 levels has a pathogenic role in the progression of nephropathy.

## Other cell cycle regulators involved in nephropathy

### p21

The CKI protein p21 has important roles in controlling cell proliferation, terminal differentiation, cellular senescence, and apoptosis [81]. p21 inhibits the cell cycle progression by binding to cyclin/CDK complexes. p21 also directly binds to proliferating cell nuclear antigen (PCNA), which inhibits the involvement of PCNA in DNA replication [98, 99]. The protein level of p21 increases in Skp2^−/−^ mouse embryo fibroblasts during the S-phase, and its degradation is low in Skp2^−/−^ cells, which suggests that p21 is a target of Skp2 degradation in the S-phase [100]. The p21 protein level is mainly controlled by transcription, but it is also subject to ubiquitin-independent and -dependent degradation [101]. In the kidneys, p21 is upregulated in the early stages of renal injury in UUO mice [102] and ATS nephropathy [95], as well as in ischemia [103] and cisplatin-treated mice [104]. p21 levels increase dramatically following growth arrest induced by the tumor suppressor protein p53 and in the early stage of differentiation [81, 105]. p21 is also induced in p53-mediated apoptosis, as the p53-dependent pathways are involved in transactivation of the p21 gene [106]. However, p21 mRNA expression was enhanced in p53-deficient mice with nephropathy, which suggests that p21 transcriptional activation occurs via a p53-independent pathway in renal damage [104]. Huge et al. also reported that the proliferation of interstitial cells, particularly myofibroblasts, was promoted in the UUO kidneys from p21^−/−^ mice compared with wild-type mice resulting in progression of renal failure, although there was no difference in the rate of interstitial cell apoptosis between these two strains. Tubular epithelial cell proliferation and apoptosis were also unchanged in the obstructed kidney from p21^−/−^ mice [91]. p21 plays a limited role in the proliferation of myofibroblasts in renal damage, and is not essential for the regulation of tubular epithelial cell proliferation or apoptosis following UUO. However, it was reported that p21 expression is increased in experimental diabetic nephropathy and inhibits mesangial cell proliferation [107]. Moreover, glomerular cell proliferation is significantly increased in glomerulonephritis in p21^−/−^ mice [108]. Taken together, these results indicate that p21 regulates the proliferation of myofibroblasts and glomerular cells in nephropathy.

### p57

The CKI protein p57 inhibits cell cycle progression into the S-phase. Overexpression of p57 induces G1-phase arrest [109, 110] and is implicated in cell cycle exit accompanying terminal differentiation [111, 112]. p57 is constitutively expressed in terminally differentiated normal mature podocytes [113, 114]. In glomerular diseases, p57 expression is decreased in podocytes, allowing mature podocytes to proliferate and acquire an immature phenotype in response to renal injury. In the ATS model, which is associated with podocyte injury, p57 expression is markedly decreased in proliferating podocytes [115]. However, the p57 protein level remains unchanged during differentiation in cultured podocytes. These properties suggest that p57 controls the proliferation of mature podocytes in nephropathy.

### p53

p53 is associated with cell proliferation, DNA repair, maintenance of DNA integrity, and apoptosis [116]. p53 regulates the induction of p21 and growth-arrested DNA damage protein 45 (GADD45) to control cell replication [117, 118]. p53 mRNA expression increases rapidly after UUO. p53 induces apoptosis of severely damaged tubular cells to limit renal damage [102]. However, tubular apoptosis after UUO is also mediated by p53-independent pathways [119].

## Perspectives

The number of patients with end-stage renal disease requiring renal replacement therapy is steadily increasing worldwide. However, the most effective therapies for this devastating disease are dialysis or kidney transplantation. Therefore, it is important to develop novel molecular targets for chronic kidney disease and avoid its progression to end-stage renal disease. Considering the results of that reports described above, it seems likely that proteasome inhibitors have some effects on Skp2-dependent protein degradation and may offer a new therapeutic drug for nephropathy, such as kidney obstruction. It has been reported that renal fibrosis is ameliorated by proteasome inhibitors in rat obstructive nephropathy [120]. Therefore, Neubert et al. [121] suggested that proteasome inhibitors are effective for treatment of nephropathy, and Pujols et al. [92] reported that a proteasome inhibitor could reduce proliferation, collagen production, and inflammatory responses in nasal fibroblasts. However, proteasome inhibitors reportedly show severe side effects because they accumulate many proteins by inhibition of proteasome-mediated degradation [122]. A described above, renal damage in UUO kidneys, including interstitial fibrosis, is markedly attenuated in Skp2^−/−^ mice compared with wild-type mice. The decreased tubular epithelial cell proliferation and reduced tubule dilation may effect the inhibition of EMT [22, 34–36] in the UUO kidneys of Skp2^−/−^ mice. We suggest that the progression of renal damage is stopped at an early stage by Skp2 deletion, reducing the extent of renal fibrosis in UUO kidneys of Skp2^−/−^ mice. Cks1 also increases p27 degradation in the early stage of renal damage, and Skp2 and Cks1 promote p27 degradation selectively in a collaborative manner. Therefore, we think an inhibitor for SCF-Skp2/Cks1 E3 ligase will offer a specific therapeutic target for renal injury and is likely to inhibit the progression of nephropathy.

## References

[1] Morgan DO (1995). Principles of CDK regulation. Nature.

[2] Sherr CJ, Roberts JM (1995). Inhibitors of mammalian G1 cyclin-dependent kinases. Genes Dev.

[3] Nourse J, Firpo E, Flanagan WM (1994). Interlukin-2-mediated elimination of the p27kip1 cyclin-dependent kinases inhibitor prevented by rapamycin. Nature.

[4] Polyak K, Lee MH, Erdjument-Bromage H (1994). Cloning of p27kip1, a cyclin-dependent kinases inhibitor and potential mediator of extracellular antimitogenic signals. Cell.

[5] Weissman AM (1997). Regulating protein degradation by ubiquitination. Immunol Today.

[6] Hershko A, Ciechanover A (1998). The ubiquitin system. Annu Rev Biochem.

[7] Carrano AC, Eytan E, Hershko A (1999). SKP2 is required for ubiquitin-mediated degradation of the CDK inhibitor p27. Nat Cell Biol.

[8] Tsvetkov LM, Yeh KH, Lee SJ (1999). p27(Kip1) ubiquitination and degradation is regulated by the SCF(Skp2) complex through phosphorylated Thr187 in p27. Curr Biol.

[9] Spruck C, Strohmaier H, Watson M (2001). A CDK-independent function of mammalian Cks1: targeting of SCF (Skp2) to the CDK inhibitor p27Kip1. Mol Cell.

[10] Ganoth D, Bornstein G, Ko TK (2001). The cell-cycle regulatory protein Cks1 is required for SCF-Skp2-mediated ubiquitinylation of p27. Nat Cell Biol.

[11] Nakayama K, Nagahama H, Minamishima YA (2000). Targeted disruption of Skp2 results in accumulation of cyclin E and p27kip1, polyploidy and centrosome overduplication. EMBO J.

[12] Couser WG, Johnson RJ (1994). Mechanisms of progressive renal disease in glomerulonephritis. Am J Kidney Dis.

[13] Gobe GC, Axelsen RA (1987). Genesis of renal tubular atrophy in experimental hydronephrosis in the rat. Role of apoptosis. Lab Invest.

[14] Walton G, Buttyan R, Garcia Montes E (1992). Renal growth factor expression during the early phase of experimental hydronephrosis. J Urol.

[15] Klahr S, Morrissey J (2002). Obstructive nephropathy and renal fibrosis. Am J Physiol Renal Physiol.

[16] Fukasawa H, Yamamoto T, Togawa A (2006). Ubiquitin-dependent degradation of SnoN and Ski is increased in renal fibrosis induced by obstructive injury. Kidney Int.

[17] Schreiner GF, Harris KP, Purkerson ML (1988). Immunological aspects of acute ureteral obstruction: immune cell infiltrate in the kidney. Kidney Int.

[18] Ricardo SD, Levinson ME, DeJoseph MR (1996). Expression of adhesion molecules in rat renal cortex during experimental hydronephrosis. Kidney Int.

[19] Chevalier RL, Forbes MS, Thornhill BA (2009). Ureteral obstruction as a model of renal interstitial fibrosis and obstructive nephropathy. Kidney Int.

[20] Suzuki S, Fukasawa H, Kitagawa K (2007). Renal damage in obstructive nephropathy is decreased in Skp2-deficient mice. Am J Pathol.

[21] Misaki T, Yamamoto T, Suzuki S (2009). Decrease in tumor necrosis factor-alpha receptor-associated death domain results from ubiquitin-dependent degradation in obstructive renal injury in rats. Am J Pathol.

[22] Yamashita S, Maeshima A, Nojima Y (2005). Involvement of renal progenitor tubular cells in epithelial-to-mesenchymal transition in fibrotic rat kidneys. J Am Soc Nephrol.

[23] Liu Y (2004). Epithelial to mesenchymal transition in renal fibrogenesis: pathologic significance, molecular mechanism, and therapeutic intervention. J Am Soc Nephrol.

[24] Fukasawa H, Fujigaki Y, Yamamato T (2012). Protein degradation by the ubiquitin-proteasome pathway and organ fibrosis. Curr Med Chem.

[25] Yamamoto T, Noble NA, Miller DE (1994). Sustained expression of TGF-beta1 underlies development of progressive kidney fibrosis. Kidney Int.

[26] Watanabe T, Yamamoto T, Ikegaya N (2002). Transforming growth factor-beta receptors in self-limited vs. chronic progressive nephropathy in rats. J Pathol.

[27] Fukasawa H, Yamamoto T, Suzuki H (2004). Treatment with anti-TGF-beta antibody ameliorates chronic progressive nephritis by inhibiting Smad/TGF-beta signaling. Kidney Int.

[28] Ng YY, Huang TP, Yang WC (1998). Tubular epithelial-myofibroblast transdifferentiation in progressive tubulointerstitial fibrosis in 5/6 nephrectomized rats. Kidney Int.

[29] Sinuani I, Weissgarten J, Beberashvili I (2009). The cyclin kinase inhibitor p57kip2 regulates TGF-beta-induced compensatory tubular hypertrophy: effect of the immunomodulator AS101. Nephrol Dial Transplant.

[30] Wolf G, Schroeder R, Thaiss F (1998). Glomerular expression of p27Kip1 in diabetic db/db mouse: role of hyperglycemia. Kidney Int.

[31] Awazu M, Omori S, Ishikura K (2003). The lack of cyclin kinase inhibitor p27(Kip1) ameliorates progression of diabetic nephropathy. J Am Soc Nephrol.

[32] Border WA, Ruoslahti E (1992). Transforming growth factor-beta in disease: the dark side of tissue repair. J Clin Invest.

[33] Eddy AA (1996). Molecular insights into renal interstitial fibrosis. J Am Soc Nephrol.

[34] Border WA, Noble NA (1994). Transforming growth factor beta in tissue fibrosis. N Engl J Med.

[35] Okada H, Danoff TM, Kalluri R (1997). Early role of Fsp1 in epithelial-mesenchymal transformation. Am J Physiol.

[36] Fan JM, Ng YY, Hill PA (1999). Transforming growth factor-beta regulates tubular epithelial-myofibroblast transdifferentiation in vitro. Kidney Int.

[37] Robert LC (2006). Obstructive nephropathy: towards biomarker discovery and gene therapy. Nat Clin Pract Nephrol.

[38] García-Sánchez O, López-Hernández FJ, López-Novoa JM (2010). An integrative view on the role of TGF-beta in the progressive tubular deletion associated with chronic kidney disease. Kidney Int.

[39] Yu L, Hébert MC, Zhang YE (2002). TGF-beta receptor-activated p38 MAP kinase mediates Smad-independent TGF-beta responses. EMBO J.

[40] Dai C, Yang J, Liu Y (2003). Transforming growth factor-beta1 potentiates renal tubular epithelial cell death by a mechanism independent of Smad signaling. J Biol Chem.

[41] Ohashi N, Yamamoto T, Uchida C (2005). Transcriptional induction of Smurf2 ubiquitin ligase by TGF-β. FEBS Lett.

[42] Fukasawa H, Yamamato T, Kitagawa M (2008). Regulation of TGF-beta signaling by Smads and its roles in tissue fibrosis. Curr Signal Trunsduct Ther.

[43] Böttinger EP, Bitzer M (2002). TGF-beta signaling in renal disease. J Am Soc Nephrol.

[44] Lin H, Wang D, Wu T (2011). Blocking core fucosylation of TGF-β1 receptors downregulates their functions and attenuates the epithelial-mesenchymal transition of renal tubular cells. Am J Physiol Renal Physiol.

[45] Derynck R, Zhang Y, Feng XH (1998). Smads: transcriptional activators of TGF-beta responses. Cell.

[46] Togawa A, Yamamoto T, Suzuki H (2003). Ubiquitin-dependent degradation of Smad2 is increased in the glomeruli of rats with anti-thymocyte serum nephritis. Am J Pathol.

[47] Wang W, Koka V, Lan HY (2005). Transforming growth factor-beta and Smad signalling in kidney diseases. Nephrology.

[48] Fukasawa H, Yamamoto T, Togawa A (2004). Down-regulation of Smad7 expression by ubiquitin-dependent degradation contributes to renal fibrosis in obstructive nephropathy in mice. Proc Natl Acad Sci USA.

[49] Oh SP, Seki T, Goss KA (2000). Activin receptor-like kinase 1 modulates transforming growth factor-beta 1 signaling in the regulation of angiogenesis. PNAS.

[50] Wang W, Ungermannova D, Jin J (2004). Negative regulation of SCFSkp2 ubiquitin ligase by TGF-beta signaling. Oncogene.

[51] Liu W, Wu G, Li W (2007). Cdh1-anaphase-promoting complex targets Skp2 for destruction in transforming growth factor beta-induced growth inhibition. MCB.

[52] Hu D, Liu W, Wu G (2011). Nuclear translocation of Skp2 facilitates its destruction in response to TGFβ signaling. Cell Cycle.

[53] He KL, Ting AT (2002). A20 inhibits tumor necrosis factor (TNF) alpha-induced apoptosis by disrupting recruitment of TRADD and RIP to the TNF receptor 1 complex in Jurkat T cells. Mol Cell Biol.

[54] Misseri R, Meldrum DR, Dinarello CA (2005). TNF-alpha mediates obstruction-induced renal tubular cell apoptosis and proapoptotic signaling. Am J Physiol Renal Physiol.

[55] Meldrum KK, Metcalfe P, Leslie JA (2006). TNF-alpha neutralization decreases nuclear factor-kappaB activation and apoptosis during renal obstruction. J Surg Res.

[56] Meldrum KK, Misseri R, Metcalfe P (2007). TNF-alpha neutralization ameliorates obstruction-induced renal fibrosis. Am J Physiol Regul Integr Comp Physiol.

[57] Ramesh G, Reeves WB (2003). TNFR2-mediated apoptosis and necrosis in cisplatin-induced acute renal failure. Am J Physiol Renal Physiol.

[58] Bharat BA (2003). Signaling pathways of the superfamily: a double-edged sword. Immunology.

[59] Gioacchino N, Liv MIA (2008). A birthday gift for TRADD. Nat Immunol.

[60] Wang D, Montgomery RB, Schmidt LJ (2009). Reduced tumor necrosis factor receptor-associated death domain expression is associated with prostate cancer progression. Cancer Res.

[61] Hsu H, Shu HB, Pan MG (1996). TRADD-TRAF2 and TRADD-FADD interactions define two distinct TNF receptor 1 signal transduction pathways. Cell.

[62] Rothe M, Sarma V, Dixit VM (1995). TRAF2-mediated activation of NF-kappa B by TNF receptor 2 and CD40. Science.

[63] Guo G, Morrissey J, McCracken R (1999). Role of TNFR1 and TNFR2 receptors in tubulointerstitial fibrosis of obstructive nephropathy. Am J Physiol.

[64] Weiss T, Grell M, Siemienski K (1998). TNFR80-dependent enhancement of TNFR60-induced cell death is mediated by TNFR-associated factor 2 and is specific for TNFR60. J Immunol.

[65] Mukhopadhyay A, Suttles J, Stout RD (2001). Genetic deletion of the tumor necrosis factor receptor p60 or p80 abrogates ligand-mediated activation of nuclear factor-kappa B and of mitogen-activated protein kinases in macrophages. J Biol Chem.

[66] Collins T (1993). Endothelial nuclear factor-kappa B and the initiation of the atherosclerotic lesion. Lab Invest.

[67] Baeuerle PA, Henkel T (1994). Function and activation of NF-kappa B in the immune system. Annu Rev Immunol.

[68] Tak PP, Firestein GS (2001). NF-κB: a key role in inflammatory disease. J Clin Invest.

[69] Ruiz-Ortega M, Bustos C, Hernández-Presa MA (1998). Angiotensin II participates in mononuclear cell recruitment in experimental immune complex nephritis through nuclear factor-kappa B activation and monocyte chemoattractant protein-1 synthesis. J Immunol.

[70] Pocock J, Gómez-Guerrer C, Harendza S (2003). Differential activation of NF-κB, AP-1, and C/EBP in endotoxin-tolerant rats: mechanisms for in vivo regulation of glomerular RANTES/CCL5 expression. J Immunol.

[71] Hayden MS, Ghosh S (2004). Signaling to NF-kappaB. Genes Dev.

[72] Cao Y, Bonizzi G, Seagroves TN (2001). IKKalpha provides an essential link between RANK signaling and cyclin D1 expression during mammary gland development. Cell.

[73] Morrissey JJ, Klahr S (1997). Rapid communication. Enalapril decreases nuclear factor kappa B activation in the kidney with ureteral obstruction. Kidney Int.

[74] Schneider G, Saur D, Siveke JT (2006). IKK alpha controls p52/RelB at the skp2 gene promoter to regulate G1- to S-phase progression. EMBO J.

[75] Barré B, Perkins ND (2010). The Skp2 promoter integrates signaling through the NF-kappaB, p53, and Akt/GSK3beta pathways to regulate autophagy and apoptosis. Mol Cell.

[76] Suzuki S, Fukasawa H, Misaki T (2011). Up-regulation of Cks1 and Skp2 with TNFα/NF-κB signaling in chronic progressive nephropathy. Genes Cells.

[77] Frescas D, Pagano M (2008). Deregulated proteolysis by the F-box proteins SKP2 and beta-TrCP: tipping the scales of cancer. Nat Rev Cancer.

[78] Sutterlüty H, Chatelain E, Marti A (1999). p45SKP2 promotes p27Kip1 degradation and induces S phase in quiescent cells. Nat Cell Biol.

[79] Kamura T, Hara T, Matsumoto M (2004). Cytoplasmic ubiquitin ligase KPC regulates proteolysis of p27(Kip1) at G1 phase. Nat Cell Biol.

[80] Hattori T, Isobe T, Abe K (2007). Pirh2 Promotes Ubiquitin-Dependent Degradation of the CDK Inhibitor p27^*Kip1*^. Cancer Res.

[81] Sherr CJ, Roberts JM (1999). CDK inhibitors: positive and negative regulators of G1-phase progression. Genes Dev.

[82] Elledge SJ, Winston J, Harper JW (1996). A question of balance: the role of cyclin-kinase inhibitors in development and tumorigenesis. Trends Cell Biol.

[83] Coats S, Flanagan WM, Nourse J (1996). Requirement of p27^kip1^ for restriction point control of the fibroblast cell cycle. Science.

[84] Shankland SJ, Pippin J, Flanagan M (1997). Mesangial cell proliferation mediated by PDGF and bFGF is determined by levels of the cyclin kinase inhibitor p27^kip1^. Kidney Int.

[85] Loda M, Cukor B, Tam SW (1997). Increased proteasome-dependent degradation of the cyclin-dependent kinase inhibitor p27 in aggressive colorectal carcinomas. Nat Med.

[86] Esposito V, Baldi A, De Luca A (1997). Prognostic role of the cyclin-dependent kinase inhibitor p27 in non-small cell lung cancer. Cancer Res.

[87] Steeg PS, Abrams JS (1997). Cancer prognostics: past, present and p27. Nat Med.

[88] Gerth JH, Kriegsmann J, Trinh TT (2002). Induction of p27^KIP1^ after unilateral ureteral obstruction is independent of angiotensin II. Kidney Int.

[89] Schaefer L, Macakova K, Raslik L (2002). Absence of decorin adversely influences tubulointerstitial fibrosis of the obstructed kidney by enhanced apoptosis and increased inflammatory reaction. Am J Pathol.

[90] Ophascharoensuk V, Fero ML, Hughes J (1998). The cyclin-dependent kinases inhibitor p27^kip1^ safeguards against inflammatory injury. Nat Med.

[91] Hughes J, Brown P, Shankland SJ (1999). Cyclin kinase inhibitor p21CIP1/WAF1 limits interstitial cell proliferation following ureteric obstruction. Am J Physiol.

[92] Pujols L, Fernández-Bertolín L, Fuentes-Prado M (2012). Proteasome inhibition reduces proliferation, collagen expression, and inflammatory cytokine production in nasal mucosa and polyp fibroblasts. J Pharmacol Exp Ther.

[93] Zhou H, Kato A, Yasuda H (2004). The induction of cell cycle regulatory and DNA repair proteins in cisplatin-induced acute renal failure. Toxicol Appl Pharmacol.

[94] Ibrahim HN, Hostetter TH (1997). Diabetic nephropathy. J Am Soc Nephrol.

[95] Chiara M, Menegatti E, Di Simone D (2004). Mycophenolate mofetil and roscovitine decrease cyclin expression and increase p27(kip1) expression in anti Thy1 mesangial proliferative nephritis. Clin Exp Immunol.

[96] Shankland SJ, Hugo C, Coats SR (1996). Changes in cell-cycle protein expression during experimental mesangial proliferative glomerulonephritis. Kidney Int.

[97] Suzuki S, Fukasawa H, Misaki T (2012). The amelioration of renal damage in Skp2-deficient mice canceled by p27 Kip1 deficiency in Skp2^−/−^ p27^−/−^ mice. PLoS One.

[98] Li R, Waga S, Hannon GJ (1994). Differential effects by the p21 CDK inhibitor on PCNA-dependent DNA replication and repair. Nature.

[99] Chen J, Jackson PK, Kirschner MW (1995). Separate domains of p21 involved in the inhibition of Cdk kinase and PCNA. Nature.

[100] Bornstein G, Bloom J, Sitry-Shevah D (2003). Role of the SCFSkp2 ubiquitin ligase in the degradation of p21Cip1 in S phase. J Biol Chem.

[101] Sheaff RJ, Singer JD, Swanger J (2000). Proteasomal turnover of p21Cip1 does not require p21Cip1 ubiquitination. Mol Cell.

[102] Morrissey JJ, Ishidoya S, McCrachen R (1996). Control of p53 and p21 (WAF1) expression during unilateral ureteral obstruction. Kidney Int Suppl.

[103] Megyesi J, Udvarhelyi N, Safirstein RL (1996). The p53-independent activation of transcription of p21 WAF1/CIP1/SDI1 after acute renal failure. Am J Physiol.

[104] Zhou H, Fujigaki Y, Kato A (2006). Inhibition of p21 modifies the response of cortical proximal tubules to cisplatin in rats. Am J Physiol Renal Physiol.

[105] Waga S, Hannon GJ, Beach D (1994). The p21 inhibitor of cyclin-dependent kinases controls DNA replication by interaction with PCNA. Nature.

[106] El-Deiry WS, Harper JW, O’Connor PM (1994). WAF1/CIP1 is induced in p53-mediated G_1_ arrest and apoptosis. Cancer Res.

[107] Kuan CJ, al-Douahji M, Shankland SJ (1998). The cyclin kinase inhibitor p21WAF1, CIP1 is increased in experimental diabetic nephropathy: potential role in glomerular hypertrophy. J Am Soc Nephrol.

[108] Kim YG, Alpers CE, Brugarolas J (1999). The cyclin kinase inhibitor p21CIP1/WAF1 limits glomerular epithelial cell proliferation in experimental glomerulonephritis. Kidney Int.

[109] Lee MH, Reynisdóttir I, Massagué J (1995). Cloning of p57KIP2, a cyclin-dependent kinase inhibitor with unique domain structure and tissue distribution. Genes Dev.

[110] Matsuoka S, Edwards MC, Bai C (1995). p57KIP2, a structurally distinct member of the p21CIP1 Cdk inhibitor family, is a candidate tumor suppressor gene. Genes Dev.

[111] Zhang P, Wong C, DePinho RA (1998). Cooperation between the Cdk inhibitors p27(KIP1) and p57(KIP2) in the control of tissue growth and development. Genes Dev.

[112] Lovicu FJ, McAvoy JW (1999). Spatial and temporal expression of p57(KIP2) during murine lens development. Mech Dev.

[113] Nagata M, Nakayama K, Terada Y (1998). Cell cycle regulation and differentiation in the human podocyte lineage. Am J Pathol.

[114] Shankland SJ, Eitner F, Hudkins KL (2000). Differential expression of cyclin-dependent kinase inhibitors in human glomerular disease: role in podocyte proliferation and maturation. Kidney Int.

[115] Hiromura K, Haseley LA, Zhang P (2001). Podocyte expression of the CDK-inhibitor p57 during development and disease. Kidney Int.

[116] Hartwell LH, Kastan MB (1994). Cell cycle control and cancer. Science.

[117] El-Deiry WS, Tokino T, Velculescu VE (1993). WAF1, a potential mediator of p53 tumor suppression. Cell.

[118] Smith ML, Chen IT, Zhan Q (1994). Interaction of the p53-regulated protein Gadd45 with proliferating cell nuclear antigen. Science.

[119] Choi YJ, Mendoza L, Rha SJ (2001). Role of p53-dependent activation of caspases in chronic obstructive uropathy: evidence from p53 null mutant mice. J Am Soc Nephrol.

[120] Tashiro K, Tamada S, Kuwabara N (2003). Attenuation of renal fibrosis by proteasome inhibition in rat obstructive nephropathy: possible role of nuclear factor kappaB. Int J Mol Med.

[121] Neubert K, Meister S, Moser K (2008). The proteasome inhibitor bortezomib depletes plasma cells and protects mice with lupus-like disease from nephritis. Nat Med.

[122] Huber JM, Tagwerker A, Heininger D (2009). The proteasome inhibitor bortezomib aggravates renal ischemia-reperfusion injury. Am J Physiol Renal Physiol.

